# Primates and mouse NumtS in the UCSC Genome Browser

**DOI:** 10.1186/1471-2105-13-S4-S15

**Published:** 2012-03-28

**Authors:** Francesco Maria Calabrese, Domenico Simone, Marcella Attimonelli

**Affiliations:** 1 Dipartimento di Bioscienze, Biotecnologie e Scienze Farmacologiche, Università di Bari, Bari, 70126, Italy

## Abstract

**Background:**

NumtS (Nuclear MiTochondrial Sequences) are mitochondrial DNA sequences that, after stress events involving the mitochondrion, colonized the nuclear genome. Accurate mapping of NumtS avoids contamination during mtDNA PCR amplification, thus supplying reliable bases for detecting false heteroplasmies. In addition, since they commonly populate mammalian genomes (especially primates) and are polymorphic, in terms of presence/absence and content of SNPs, they may be used as evolutionary markers in intra- and inter-species population analyses.

**Results:**

The need for an exhaustive NumtS annotation led us to produce the Reference Human NumtS compilation, followed, as reported in this paper, by those for chimpanzee, rhesus macaque and mouse ones. Identification of NumtS inside the UCSC Genome Browser and their inter-species comparison required the design and the implementation of NumtS tracks, starting from the compilation data. NumtS retrieval through the UCSC Genome Browser, in the species examined, is now feasible at a glance.

**Conclusions:**

Analyses involving NumtS tracks, together with other genome element tracks publicly available at the UCSC Genome Browser, can provide deep insight into genome evolution and comparative genomics, thus improving studies dealing with the mechanisms that drove the generation of NumtS. In addition, the NumtS tracks constitute a useful tool in the design of mitochondrial DNA primers.

## Background

Colonization of the nuclear genome by mitochondrial DNA fragments began shortly after endosymbiosis and, while still ongoing, has laid the groundwork for a scenario composed of recombination events which generated duplications and *de novo *insertions. The integration or rather the "capture" of these fragments may occur during repair of double-strand breaks in nuclear DNA by NHEJ (Non-Homologous End-Joining) recombinations. These events arise due to the action of endogenous and exogenous agents such as ionizing radiation and are strictly dependent on the rate of double-strand breaks in nuclear DNA [1,2]. In some organisms, particularly primates, the same mitochondrial region occurs several times along the nuclear genome, showing how duplication events have driven the spread of NumtS among these species [1-3]. This is also indicated by the discrepancies in NumtS content among human and chimpanzee, although they are closely located in the phylogenetic tree. Another important feature of NumtS concerns the intra-specific polymorphic state, both in terms of presence/absence and SNP content: they may occur in homo- or heterozygosis or are absent in various individuals at specific loci. This feature makes them suitable for human and primate population analyses, since the most enriched species harbored this branch of the whole phylogenetic tree [4,5]. Although several clinical studies report data on NumtS involvement in causing heteroplasmy artefacts of mtDNA PCR amplification, no works have so far been published reporting tools allowing the NumtS browsing. NumtS content among mammalian and non-mammalian species has also been investigated, but the data published so far do not allow the indepth knowledge of the genomic rearrangements in which NumtS are involved. The construction of annotation tracks within the UCSC Genome Browser will certainly improve such investigations. Setting the human "NumtSome" catalog on hg18 build allowed the complete collection of a nuclear-mitochondrial "library" comprising more than 500 NumtS, i.e. RHNumtS.2 [6], and new compilations for other organisms have also been produced. In more detail, the same optimized protocol which allowed the detection of human NumtS, based on *in silico *hybridization between the human mtDNA reference sequence (rCRS) [7] and the human nuclear genome assembly, was applied to *Pan troglodytes *and *Mus musculus*. Our choice fell on those species which, at the time of the inspection (2010), were reported in the NCBI genome statistics pages as mammalian genomes in a complete state of assembly. In order to take into account an outgroup for the hominoid branch, the NumtS annotation in rhesus macaque (*Macaca mulatta*) was also performed, although its genome was, at the time of the study, in a draft state of assembly. As regards chimpanzee and mouse, although several studies have published the number of NumtS and their genome coverage [3-5,8,9], no data about genome positions are available. Here we report an improvement in terms of the quantity and quality of data and the complete annotations of NumtS. This kind of data for rhesus macaque have never been published. The NumtS compilations produced for the above-mentioned species allowed the design of tracks inside the UCSC Genome Browser. The growing demand for genomic details in these species, which are commonly used within the UCSC, encouraged us to publish the NumtS mouse (mm9 build) and human (hg19 build) tracks. Annotations about NumtS in the species (chimpanzee and rhesus macaque) which, in our opinion, will be extensively used, can be uploaded as NumtS custom annotation tracks from our local server. Our purpose is to provide a proper support to NumtS surveys and to facilitate comparative NumtS analyses, an aspect which has previously been carried out only in chimpanzee/human comparisons [3-5].

## Methods

### Blasting of nuclear genome versus mitochondrial genome

Bl2seq, which implements the BlastN program (release 2.2.19 of the Blast suite) thus allowing pairwise comparisons, was launched on the entire chromosomal set for each species: 24 for *Pan troglodytes *(panTro2 build), 21 for *Macaca mulatta *(rheMac2 build) and *Mus musculus *(mm9 build). The entire chromosome dataset for each build was downloaded at the following address [10]. Job executions were performed on a local server. The accession numbers of the mtDNA reference sequences used as queries are respectively NC_005943 for rhesus macaque, NC_001643 for chimpanzee and NC_005089 for mouse. The same protocol applied to the generation of the human compilation (RHNumtS.2) [6] was used for the other three species, with scoring parameters fixed as follows: 2 for match reward, -3 for mismatch penalty, -5 for gap opening, -2 for gap extension. The expected value (e-value) was fixed at 1e-03. Each fragment of each chromosome aligned with the mtDNA whose e-value was lower than the fixed threshold produced an HSP (High Scoring Pair). Each HSP was associated with a NumtS. The complete list of NumtS loci for each species was annotated in a spreadsheet (NumtS compilation).

### NumtS assembly

NumtS mapping at a distance of less than 2000 bp on a specific chromosome and corresponding to two mtDNA fragments, at most distant 2000 bp and oriented in the same direction, were combined in a single NumtS and called "assembled NumtS". This was done by spreadsheet interpolation and manual inspection, well supported by graphical display on the UCSC Genome Browser and with tools available within the Galaxy platform [11-14]. As in the generation of human assembled NumtS, the fragment-joining protocol was slightly modified for HSPs, interposed by long repetitive elements in order to compare fragments which followed the same evolutionary history, at least among primate lineage.

### NumtS track implementation

Starting from the NumtS compilation for each species, four tracks were implemented: "NumtS" and "NumtS Assembled" tracks, displaying data from the corresponding compilations; the "NumtS on mitochondrion" track, showing mapping of NumtS on the mitochondrial genome, and the "NumtS on mitochondrion with mismatches" track, showing mismatches between NumtS sequence and mitochondrial genome. Since the mitochondrial chromosome is not available in the UCSC Genome Browser for *Macaca mulatta*, the "NumtS on mitochondrion" and the "NumtS on mitochondrion with mismatches" tracks were not designed for this species.

### Creating BED and BAM tracks

**Figure 1:**
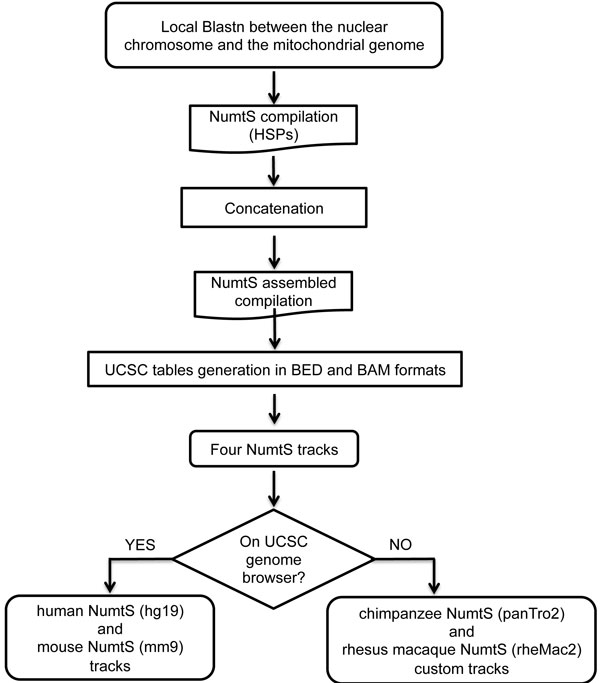
**NumtS track flowchart**. Step-by-step protocol, implemented to produce both NumtS compilations and NumtS tracks in the four species examined.

UCSC Genome Browser tracks may be produced and configured in a variety of ways, to highlight features needed. The format used for "NumtS", "NumtS Assembled" and "NumtS on mitochondrion" tracks is the Browser Extensible Data format (BED) which is a flexible way of showing genomic annotations. In-house shells and Python scripts were used to produce BED and BAM formatted data and to implement inter-NumtS track links, starting from the UCSC description pages, providing further insights when switching from the nuclear locus of a NumtS to its mitochondrial counterpart. The BAM format, the compressed binary version of the Sequence Alignment/Map (SAM) format, was used for the "NumtS on mitochondrion with mismatches" track generation. The LASTZ software [15] was used to realign each NumtS to its mitochondrial sequence, using default parameters and setting the SAM format as output. SAM files were converted to BAM format with the SAMtools package [16]. Figure 1 shows the flowchart followed to obtain the NumtS tracks starting from the Blastn, giving HSPs and passing through NumtS compilations.

### Remapping of human tracks on hg19

The revised reference human NumtS compilation (RHNumtS.2), already implemented inside the UCSC Genome Browser according to the hg18 build, was upgraded to the GRCh37 build (hg19). Genomic coordinates were converted by using the "Lift-Over" tool available through the Galaxy suite. Human hg19 compilation is available in additional file 1, RHNumtS.2 compilation.

### Comparison with previously published compilations

To compare NumtS intervals with those available in other previously published NumtS compilations, the "Lift-Over" tool, based on Lift-Over utility and Chain track, and the "Join" tool were used. They are both free and available in the Galaxy suite at UCSC Genome Browser [14].

## Results

NumtS insertions appear to have been more or less continuous over time in the lineages leading to the human genome [2-5,8]. After the RHNumtS.2, in view of the proximity between human and chimpanzee (5-7 million of years) and taking into account the level of similarity between their genomes, the NumtS comparison between them is an important step in the evolutionary study of the NumtS. Thus, by applying the same protocol used for RHNumtS.2, we have produced respectively the RPNumtS, RRNumtS and RMNumtS compilations for *Pan troglodytes, Macaca mulatta *and *Mus musculus *species. Detailed analyses of the quantity of the species-specific NumtS insertion/duplication events which occurred after the chimpanzee-human divergence are needed, to understand the underlying phylogenetic relationships. We produced the UCSC tracks with this aim in mind.

### Gathering NumtS in *Pan troglodytes*

**Figure 2:**
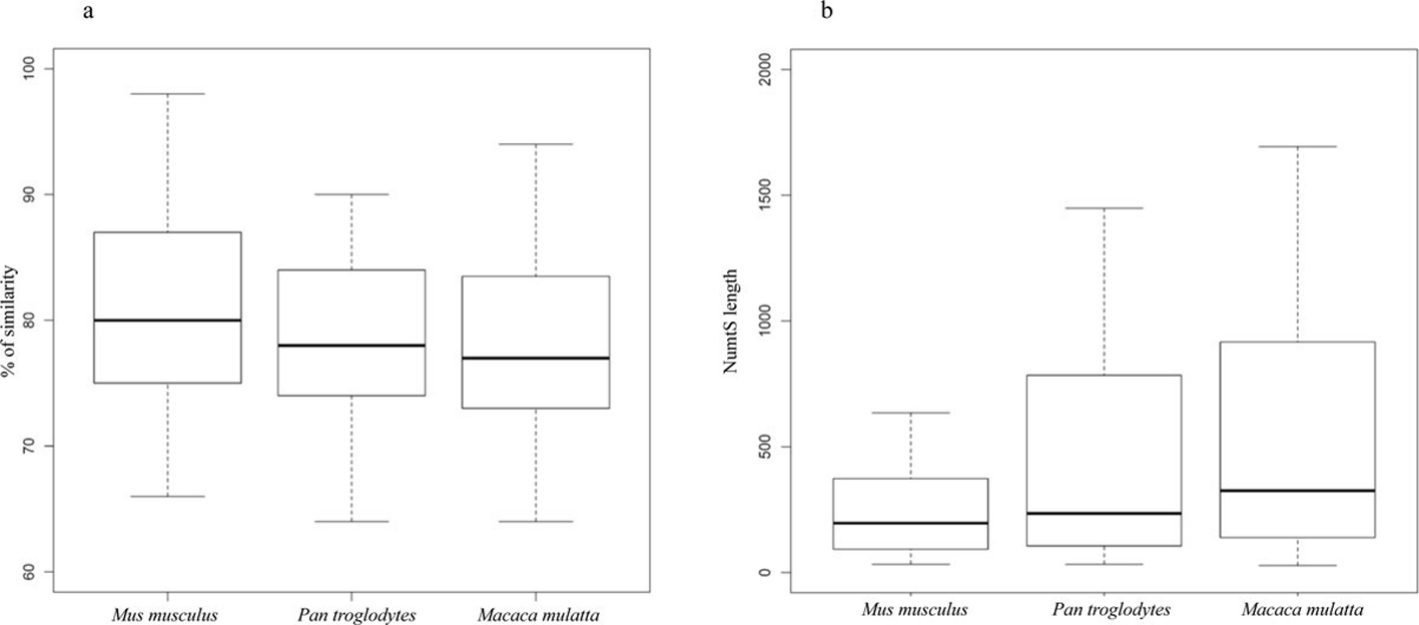
**Comparison of the box plots for NumtS lengths and similarity distributions among species examined**. **a) **Box plot based on distribution of NumtS percentage of similarity with respect to corresponding mitochondrial reference genome in three species examined. Mus musculus has the highest values. **b) **Box plots for NumtS lengths, based on mouse, chimpanzee and rhesus macaque compilations. Rhesus macaque shows highest median and maximum values.

The RPNumtS (Reference *Pan troglodytes *NumtS) compilation (additional file 2, RPNumtS compilation) annotates 776 NumtS, including 117 NumtS assembled by applying the criteria described in the Methods section. The nuclear spans range from 33 bp to 8984 bp, with a percentage of similarity ranging from 64.4 to 100 (Figure 2). An ID was assigned to each chimpanzee NumtS according to the format Ptr_NumtS_xxx, where Ptr stands for *Pan troglodytes *and xxx is a three-digit code.

### Gathering NumtS in *Macaca mulatta*

The number of HSPs returned by the similarity search analysis in rhesus macaque was 751, 113 of which were concatenated. The nuclear spans range from 28 bp to 6642 bp with a percentage of similarity ranging from 64 to 100 (Figure 2). The ID format used for the rhesus macaque NumtS was Rhm_NumtS_xxx, where Rhm stands for rhesus macaque. The RRNumtS (Reference Rhesus NumtS) compilation is available in additional file 3, RRNumtS compilation.

### Gathering NumtS in *Mus musculus*

The number of HSPs returned by the Blastn on the mouse genome is 172 of which 13 were assembled. The nuclear spans range from 33 bp to 4654 bp with a percentage of similarity ranging from 66 to 100 (Figure 2). Also for mouse the nomenclature follows the same rule as the other species: Mms_NumtS_xxx where Mms stands for *Mus musculus*. The RMNumtS (Reference Mouse NumtS) compilation is available in additional file 4, RMNumtS compilation.

### Browsing the NumtS tracks inside the UCSC Genome Browser

Data in additional files 1, 2, 3 and 4 were used to set up the "NumtS Sequence" track group on the UCSC Genome Browser ("Variation and Repeats" section) for human on hg19, chimpanzee, rhesus macaque and mouse, respectively. Implementation of the NumtS compilations on the UCSC Genome Browser for human and mouse data facilitates access to the NumtSome annotations. Once connected to UCSC [17], the presence of any NumtS in a human (hg18 or hg19 build) or murine (mm9) genomic region of interest (selected by typing its coordinates in the "position/term" search box) can be checked; alternatively, by typing a NumtS identifier in the same search box, a list of tracks available for that NumtS appears. Clicking on one of the results displays the selected item in the Genome Browser. For each NumtS, the description page reports a link which guides users in the shift from the NumtS nuclear position to its mitochondrial counterpart and vice versa. Because tracks can easily connect annotation data, they provide a very useful tool in understanding the evolutionary dynamics in which NumtS are involved and characterizing their genomic context. *Pan troglodytes *NumtS tracks, available on our local server, can be uploaded as custom annotation tracks starting from the "NumtS on mitochondrion track" with the link reported in the reference section [18]. Starting from the mitochondrial track, clicking on any NumtS shows external page providing further specifications about that NumtS (together with the others) in UCSC Genome Browser "detail page" fashion. This procedure substitutes the "NumtS Sequence" track activation described above for human and mouse tracks. For *M. mulatta *custom tracks activation, because of the lack in the rhesus macaque build of the mitochondrial genome, means that the starting link points to a nuclear location, at the same time activating the NumtS track. The link is reported in the reference section [19].

### Comparisons with previously published compilations

Other research groups have already reported NumtS overviews in primates and mouse. In order to demonstrate the improvements obtained with our approach, comparisons between our compilations and publicly available data for chimpanzee [3,5,9], rhesus macaque [3,9] and mouse [8,9] are reported here. The results are listed in additional file 5, NumtS compilations comparison. HSP number and base pair coverage on the entire genome, as well as the same features for the assembled NumtS, are given. Differences in compilation contents for the same organism are due to differences between one release and another and to the Blastn algorithm parameters chosen. The only NumtS locations available are those published in [5] concerning NumtS in *Pan troglodytes*. Merging of these annotations with our compilation shows that all the items reported in [5] are included within our dataset. Further details about the comparisons of NumtS compilations are given in additional file 6, RPNumtS annotation comparison.

## Discussion and conclusions

The above protocol produced 776, 751 and 173 HSPs in chimpanzee, rhesus macaque and mouse, respectively. These data confirm and extend the NumtS catalog for different species, published so far and summarised in [8]. To test the statistical significance of the NumtS lengths in each species, we have used a Pearson correlation matrix (additional file 7, NumtS and genome features correlation matrix), highlighting a strong correlation between genome size and total amount of NumtS. Figures 2a and 2b show that mouse NumtS are the least, the shortest but also the most highly conserved. The availability of the tracks will allow us to go into further detail on this aspect. In order to facilitate NumtS inter- and intra-species studies on the basis of our compilations, we produced the UCSC Genome Browser tracks, representing a reliable and useful way of recognizing and interrelating them with other genomic elements. Among the most important advantages linked to track implementation are i) the comparison of NumtS data with any other type of genomic element annotation through tracks in the browser, thus allowing NumtS to be mapped in syntenic regions as well as in repetitive regions; ii) the possibility of retrieving the entire set of a track data through the "Tables" section, allowing a custom NumtS database to be produced; iii) the possibility of checking for risk of co-amplification in the process of mtDNA amplification [20].

### Syntenic analyses

**Figure 3:**
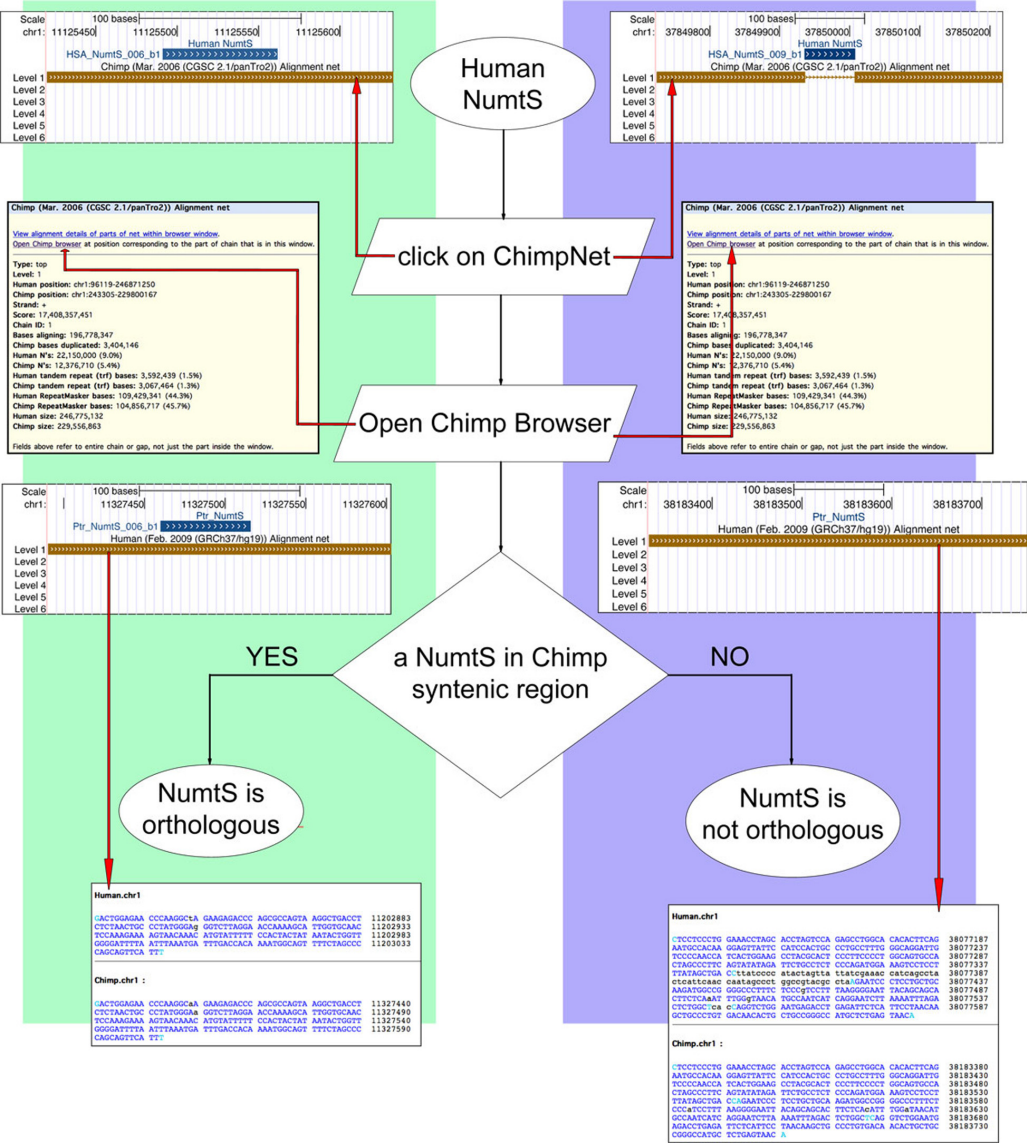
**Browsing UCSC NumtS tracks to identify orthologous NumtS**. Workflow shown assesses presence of identical NumtS in two species for which the UCSC annotation tracks were produced. Graphs show two human NumtS, (HSA_NumtS_006_b1, HSA_NumtS_009_b1) and ChimpNet track, indicating that the former is also present in chimpanzee, whereas the latter is not. Following flowchart (and clicking on links and boxes marked with red arrows), this hypothesis can be verified on Chimp Browser. Alignment provided by Net tracks provides additional evidence of presence or absence of NumtS in species examined. See additional file 8, Figure 2 screenshots for magnified versions of screenshots.

The whole UCSC Genome Browser is in fact a container of browsers for various species. It can be used to classify the NumtS of two different species as orthologous or not simply by using genome browsers for which NumtS tracks have been designed and implemented. Starting from a NumtS in one of the species, for which data in the comparative genomic section are available, and shifting to another genome browser for which the NumtS tracks have also been set up, the presence/absence of a gap in the NumtS region can identify species-specific or orthologous NumtS. As an example to demonstrate the advantage of the availability of NumtS tracks for different species, starting from the HSA_NumtS_006 in the human browser, Figure 3 shows the path to be followed to recognize its orthologous, Ptr_NumtS_006 in chimpanzee (for magnified screenshots of Figure 3, see additional file 8, Figure 2 screenshots).

### NumtS and RE content among primates and mouse genomes

**Figure 4:**
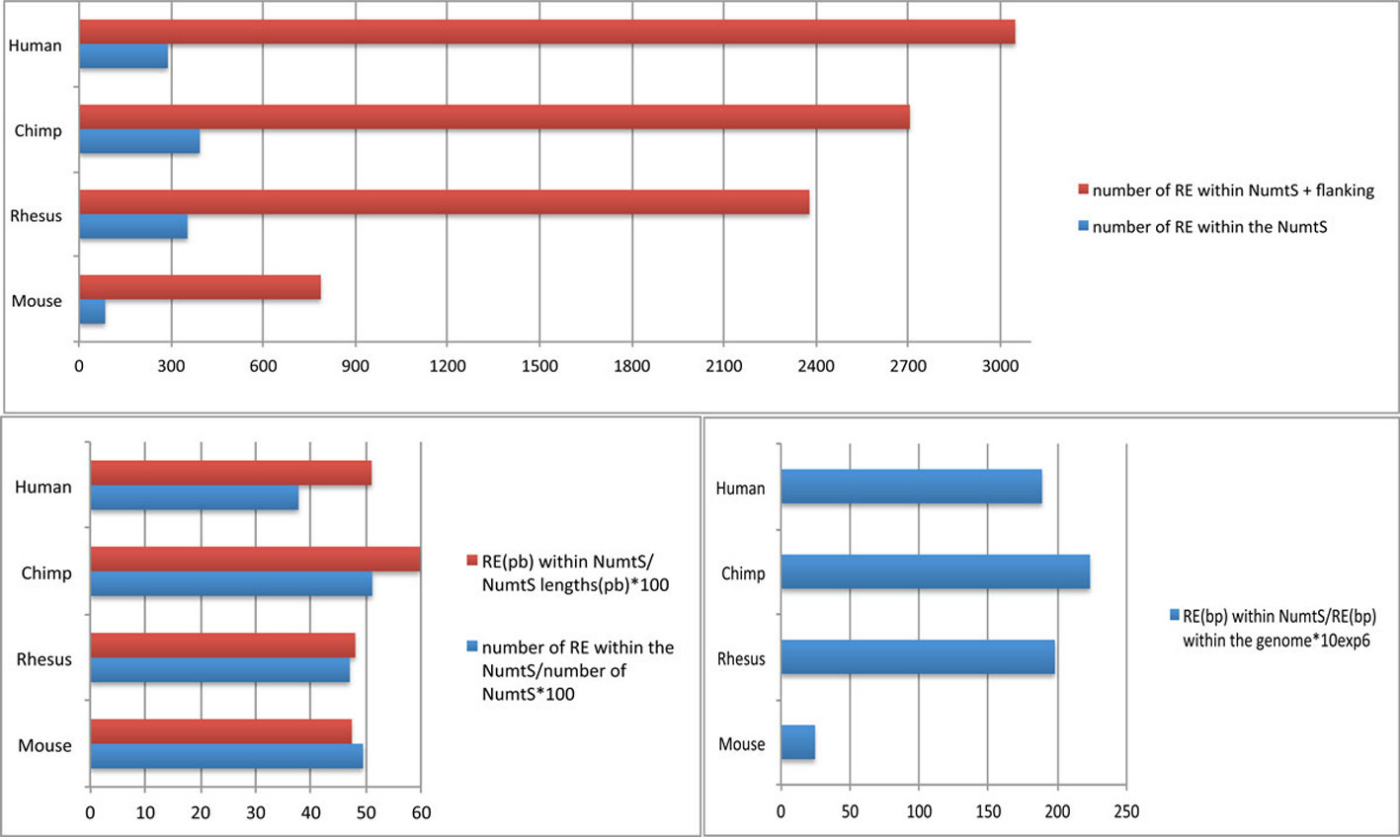
**Repeated elements within NumtS: content comparisons among species examined**. **a) **Total amount of RE within NumtS genomic intervals, with and without flanking regions (1000 bp upstream and downstream), calculated for each species. Trend in any of species examined reveals more REs in flanking regions with respect to NumtS region alone, with greater emphasis on mouse genome. **b) **Total RE content (bp and number) in NumtS loci on total NumtS length and number respectively, calculated as percentage. **c) **Percentage of RE(bp) within NumtS loci on RE content within whole genome. Although mouse genome is less populated with REs, its NumtS are populated by REs with same percentage observed in other species examined.

Figure 4 shows the statistical estimate of the relationship between NumtS and Repetitive Elements (RE). In all the considered species, as the number of RE in the regions, including the flanking region, is significantly high, NumtS insertions may occur preferentially in highly repetitive regions, with a bias in human NumtS, taking into account the fact that the chimpanzee genome has a greater quantity of NumtS. A more detailed analysis in terms of length of both NumtS and RE indicates that chimpanzee NumtS contain a higher density of RE with respect to the others. This is confirmed by the trend of the ratio between the base coverage of RE within NumtS with respect to the same value found in the entire genome (Figures 4b and 4c).

### Risk of co-amplification

**Figure 5:**
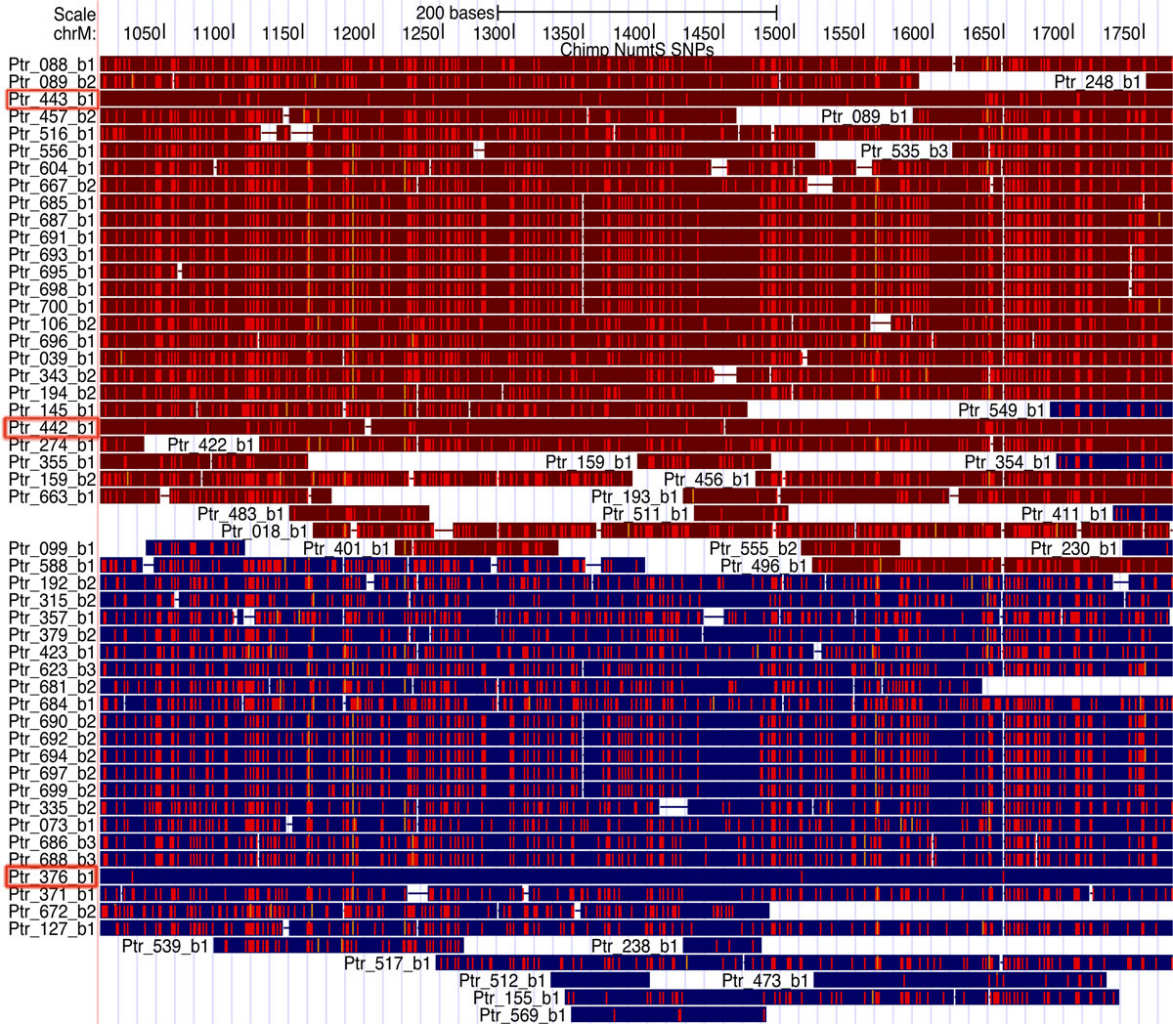
**Evidence of risk of coamplification due to NumtS**. Chimpanzee NumtS spanning within mitochondrial region from 1006 to 1771; first and second large blocks in red and blue include NumtS on minus and plus strand respectively. Red squared NumtS IDs (Ptr_NumtS _443_b1 and Ptr_NumtS _376_b1) correspond to potentially unintended nuclear regions found by Primer-Blast software, and have fewest mismatches. Red squared NumtS Ptr_NumtS _442_b1 not found by Primer-Blast software, has more mismatches.

As a result of finding pair-specific PCR primer for a chimpanzee mitochondrial region (*e.g*. range 1000-1800 in the mitochondrial genome), the Primer-Blast software [21] returns two products on potentially unintended nuclear templates for the best primer pair. Browsing the NumtS tracks proves that the two nuclear regions each contain a NumtS. Table 1 lists the NumtS sorted by score, which spans the same chimpanzee mitochondrial region, extracted from the "Ptr_NumtS on mitochondrion" track in the relative table browser. The first two highest scores are for Ptr_NumtS_376_b1 and Ptr_NumtS_443_b1, as shown in Figure 5. The third score value corresponds to Ptr_NumtS_442_b1, which contains more differences than the other two with respect to hg18 (and is thus not considered in the Primer_Blast report). The match between the data demonstrates the advantage offered by the NumtS tracks in avoiding NumtS coamplification.

**Table 1:** Chimpanzee NumtS annotation as reported in the compilation limited to the mitochondrial region 1006-1771 considered in the Primer selection example

#chrom	chromStart	chromEnd	NumtS_ID	score	strand
chrM	318	2790	Ptr_NumtS_376_b1	983	+
chrM	0	2116	Ptr_NumtS_443_b1	951	-
chrM	457	2391	Ptr_NumtS_442_b1	944	-
chrM	0	2512	Ptr_NumtS_379_b2	840	+
chrM	0	2512	Ptr_NumtS_315_b2	838	+
chrM	0	2831	Ptr_NumtS_667_b2	808	-
chrM	835	2448	Ptr_NumtS_127_b1	805	+
chrM	0	2586	Ptr_NumtS_192_b2	794	+
chrM	19	2227	Ptr_NumtS_106_b2	790	-
chrM	703	2783	Ptr_NumtS_371_b1	790	+
chrM	21	5619	Ptr_NumtS_039_b1	786	-
chrM	79	2939	Ptr_NumtS_343_b2	785	-
chrM	0	2249	Ptr_NumtS_604_b1	779	-
chrM	0	2236	Ptr_NumtS_423_b1	775	+
chrM	19	5841	Ptr_NumtS_073_b2	774	+
chrM	4	4498	Ptr_NumtS_335_b2	770	+
chrM	84	9112	Ptr_NumtS_194_b2	770	-
chrM	0	1786	Ptr_NumtS_516_b1	767	-
chrM	0	4603	Ptr_NumtS_088_b1	765	-
chrM	0	4321	Ptr_NumtS_699_b2	758	+
chrM	0	4343	Ptr_NumtS_698_b1	758	-
chrM	0	4343	Ptr_NumtS_685_b1	758	-
chrM	0	4321	Ptr_NumtS_697_b2	757	+
chrM	0	4321	Ptr_NumtS_693_b1	757	-
chrM	0	4343	Ptr_NumtS_700_b1	756	-
chrM	0	4321	Ptr_NumtS_695_b1	756	-
chrM	0	4321	Ptr_NumtS_694_b2	756	+
chrM	0	4343	Ptr_NumtS_692_b2	756	+
chrM	0	4343	Ptr_NumtS_691_b1	756	-
chrM	0	4321	Ptr_NumtS_690_b2	756	+
chrM	0	4343	Ptr_NumtS_687_b1	756	-
chrM	0	4343	Ptr_NumtS_623_b3	755	+
chrM	19	4343	Ptr_NumtS_686_b3	746	+
chrM	19	4343	Ptr_NumtS_696_b1	745	-
chrM	19	4343	Ptr_NumtS_688_b3	745	+
chrM	0	2183	Ptr_NumtS_684_b1	708	+

To conclude, as demonstrated in the above discussion, the compilations reported here represent a complete set of primate and mouse NumtS loci.

## List of abbreviations used

BAM: Binary Alignment/Map; BED: Browser Extensible Data; Bl2seq: BLAST 2 sequences; e-value: expected value; HSP: High Scoring Pairs; Mms: Mus musculus; NHEJ: Non-Homologous End-Joining; NumtS: Nuclear mitochondrial Sequences; Ptr: Pan troglodytes; rCRS: revised Cambridge Reference Sequence; RE: Repeated Elements; Rhm: Rhesus macaque; RHNumtS: Reference Human NumtS (compilation); RMNumtS: Reference Mus musculus NumtS (compilation); RPNumtS: Reference Pan troglodytes NumtS (compilation); RRNumtS: Reference Rhesus macaque NumtS (compilation); SAM: Sequence Alignment/Map.

## Competing interests

The authors declare that they have no competing interests.

## Authors' contributions

FMC carried out bioinformatics analyses and wrote the manuscript, DS and FMC designed and implemented UCSC tracks in collaboration with the UCSC staff; MA coordinated and supervised the whole project. All authors read and approved the final manuscript.

## Supplementary Material

Additional file 1 **RHNumtS.2 compilation**. Human reference NumtS compilation on hg19 build. Each line refers to a NumtS and reports NumtS ID, HSP_NumtS ID, chromosome where NumtS is located, and strand where Blast mapped NumtS ("+" if in same direction as nuclear reference sequence, "-" if in opposite direction), chromosome and mitochondrial locations. For assembled NumtS (right), detailed information on each fragment is available. Last column shows identity percentage as reported in Blast output for each HSP. Click here for file

Additional file 2 **RPNumtS compilation**. Chimpanzee reference NumtS compilation on panTro2 build. Each line refers to a NumtS and gives NumtS ID, HSP_NumtS ID, chromosome where NumtS is located and strand where Blast mapped NumtS ("+" if in same direction as nuclear reference sequence, "-" if in opposite direction), chromosome and mitochondrial locations. For assembled NumtS (right), detailed information on each fragment is available. Last column shows identity percentage as reported in Blast output for each HSP. Click here for file

Additional file 3 **RRNumtS compilation**. *Macaca mulatta *reference NumtS compilation on rheMac2 build. Each line refers to a NumtS and gives NumtS ID, HSP_NumtS ID, chromosome where NumtS is located and strand where Blast mapped NumtS ("+" if in same direction as nuclear reference sequence, "-" if in opposite direction), chromosome and mitochondrial locations. For assembled NumtS (right), detailed information on each fragment is available. Last column shows identity percentage as reported in Blast output for each HSP. Click here for file

Additional file 4 **RMNumtS compilation**. *Mus musculus *reference NumtS compilation on mm9 build. Each line refers to a NumtS and gives NumtS ID, HSP_NumtS ID, chromosome where the NumtS is located and strand where Blast mapped NumtS ("+" if in same direction as nuclear reference sequence, "-" if in opposite direction), chromosome and mitochondrial locations. For assembled NumtS (right), detailed information on each fragment is available. Last column shows identity percentage as reported in Blast output for each HSP. Click here for file

Additional file 5 **NumtS compilation comparisons**. Differences in NumtS number and length (bp) in same species are due to different assembly and differences in parameters used to launch in *silico *hybridization. Discrepancies observed in both chimpanzee and rhesus macaque data between our results and those reported in [3], where the same assemblies and same e-value were used, cannot be explained, because information on Blast running in [3] is not complete. Instead, trend of differences observed in mouse match assembly time and parameters. Click here for file

Additional file 6 **RPNumtS annotation comparisons**. Comparison were made by applying "Join" tool (UCSC Genome Browser) to compare our NumtS genomic intervals with those extracted from only previously published NumtS set available. Since this starting dataset contains orthologous NumtS only (human/chimpanzee orthologous), resulting output is partial. Data reported in [5] exactly match our data. Click here for file

Additional file 7 **NumtS and genome features correlation matrix**. A Pearson correlation matrix was calculated to test significance of NumtS lengths in species examined, with respect to relative genome lengths. Click here for file

Additional file 8 **Figure 2 screenshots**. This file provides magnified versions of screenshots shown in Figure 2. Click here for file
