# Supplementary material for: Primates and mouse NumtS in the UCSC Genome Browser
Source: BMC Bioinformatics. 2012 Mar 28;13(Suppl 4):S15. doi: 10.1186/1471-2105-13-S4-S15 (PMC3314570; doi:10.1186/1471-2105-13-S4-S15)
Supplement: Additional file 5 — NumtS compilation comparisons. Differences in NumtS number and length (bp) in same species are due to different assembly and differences in parameters used to launch in silico hybridization. Discrepancies observed in both chimpanzee and rhesus macaque data between our results and those reported in [3], where the same assemblies and same e-value were used, cannot be explained, because information on Blast running in [3] is not complete. Instead, trend of differences observed in mouse match assembly time and parameters. [file 1471-2105-13-S4-S15-S5.pdf]

Species	Reference	Genome assembly	HSPs (number and size)	Assembled NumtS (number and size)	Blastn e-value
Pan troglodytes	[5]	panTro1	n.d.	469 (165892)	0.001
Pan troglodytes	[3]	panTro2	1128(298937)	547 (569329)	0.001
Pan troglodytes	[8]	panTro2	1065(294682)	n.d.	0.0001
Pan troglodytes	this paper	panTro2	776(539672)	700 (616997)	0.001
Mus musculus	[9]	mm4	n.d.(53453)	n.d.	10exp-50<E<10exp-5
Mus musculus	[8]	mm9	137(37670)	n.d.	0.0001
Mus musculus	this paper	mm9	172(59678)	148 (66038)	0.001
Macaca mulatta	[3]	rheMac2	860(264929)	434(454,441)	0.001
Macaca mulatta	[8]	rheMac2	804(261622)	n.d.	0.0001
Macaca mulatta	this paper	rheMac2	751(523156)	553(602465)	0.001

n.d. stands for not determined
