# Supplementary material for: Primates and mouse NumtS in the UCSC Genome Browser
Source: BMC Bioinformatics. 2012 Mar 28;13(Suppl 4):S15. doi: 10.1186/1471-2105-13-S4-S15 (PMC3314570; doi:10.1186/1471-2105-13-S4-S15)
Supplement: Additional file 7 — NumtS and genome features correlation matrix. A Pearson correlation matrix was calculated to test significance of NumtS lengths in species examined, with respect to relative genome lengths. [file 1471-2105-13-S4-S15-S7.pdf]

	genome_length	NumtS_number	Σ_NumtS_length	Σ_NumtS_length /genome_length*10.9	mean_NumtS_length /genome_length*10.9
**genome_length**	10.00	0.84	0.84	0.79	0.78
**NumtS_number**	0.84	10.00	0.99	0.99	0.98
**Σ_NumtS_length**	0.84	0.99	10.00	0.99	0.99
**Σ_NumtS_length /genome_length*10.9**	0.79	0.99	0.99	10.00	0.99
**mean_NumtS_length /genome_length*10.9**	0.78	0.98	0.99	0.99	10.00
