# Supplementary material for: Primates and mouse NumtS in the UCSC Genome Browser
Source: BMC Bioinformatics. 2012 Mar 28;13(Suppl 4):S15. doi: 10.1186/1471-2105-13-S4-S15 (PMC3314570; doi:10.1186/1471-2105-13-S4-S15)
Supplement: Additional file 8 — Figure 2 screenshots. This file provides magnified versions of screenshots shown in Figure 2. [file 1471-2105-13-S4-S15-S8.pdf]

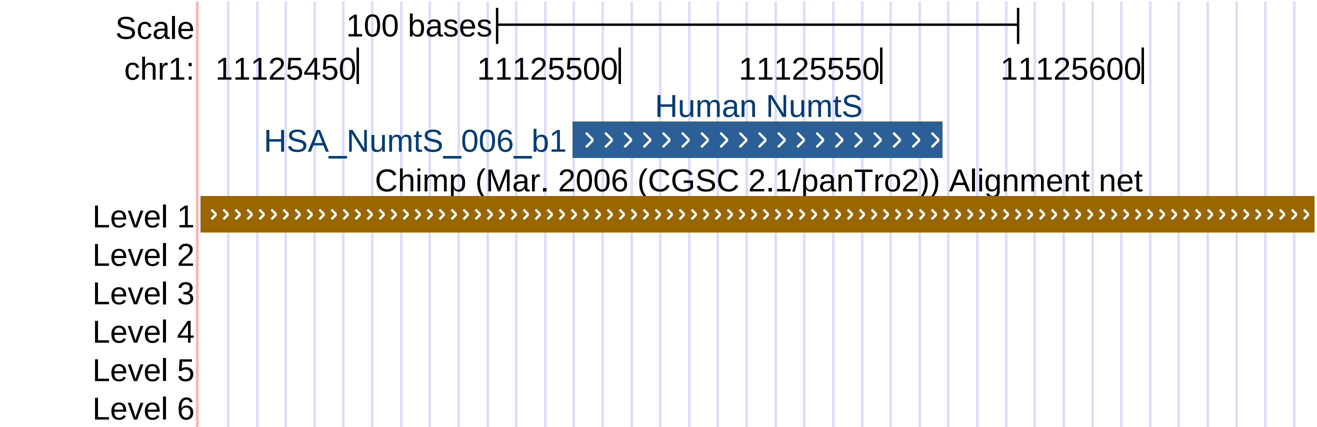

Scale
100 bases
chr1:
11125450
11125500
11125550
11125600
Human NumtS
HSA_NumtS_006_b1
Chimp (Mar. 2006 (CGSC 2.1/panTro2)) Alignment net
Level 1
Level 2
Level 3
Level 4
Level 5
Level 6

Chimp (Mar. 2006 (CGSC 2.1/panTro2)) Alignment net

View alignment details of parts of net within browser window.
Open Chimp browser at position corresponding to the part of chain that is in this window.

Type: top
Level: 1
Human position: chr1:96119-246871250
Chimp position: chr1:243305-229800167
Strand: +
Score: 17,408,357,451
Chain ID: 1
Bases aligning: 196,778,347
Chimp bases duplicated: 3,404,146
Human N's: 22,150,000 (9.0%)
Chimp N's: 12,376,710 (5.4%)
Human tandem repeat (trf) bases: 3,592,439 (1.5%)
Chimp tandem repeat (trf) bases: 3,067,464 (1.3%)
Human RepeatMasker bases: 109,429,341 (44.3%)
Chimp RepeatMasker bases: 104,856,717 (45.7%)
Human size: 246,775,132
Chimp size: 229,556,863

Fields above refer to entire chain or gap, not just the part inside the window.

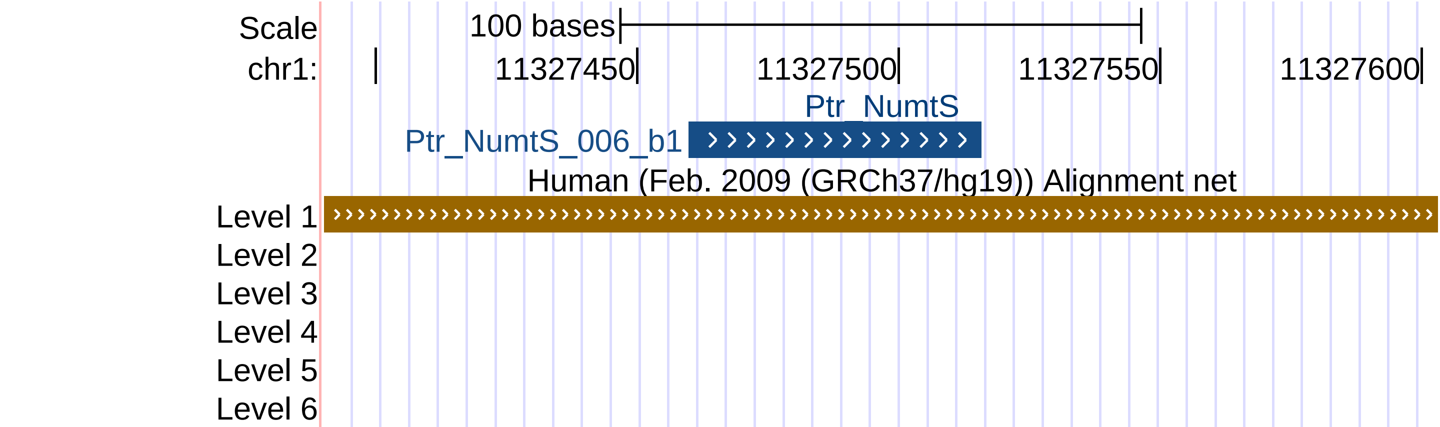
Scale
100 bases
chr1:
11327450
11327500
11327550
11327600
Ptr_NumtS
Ptr_NumtS_006_b1
Human (Feb. 2009 (GRCh37/hg19)) Alignment net
Level 1
Level 2
Level 3
Level 4
Level 5
Level 6

Human.chr1

```
GACTGGAGAA CCCAAGGCtA GAAGAGACCC AGCGCCAGTA AGGCTGACCT  11202883
CTCTAACTGC CCTATGGGAg GGTCTTAGGA ACCAAAAGCA TTGGTGCAAC  11202933
TCCAAAGAAA AGTAACAAAC ATGTATTTTT CCACTACTAT AATACTGGTT  11202983
GGGGATTTTA ATTTAAATGA TTTGACCACA AAATGGCAGT TTTCTAGCCC  11203033
CAGCAGTTCA TTT
```

Chimp.chr1 :

```
GACTGGAGAA CCCAAGGCaA GAAGAGACCC AGCGCCAGTA AGGCTGACCT  11327440
CTCTAACTGC CCTATGGGAa GGTCTTAGGA ACCAAAAGCA TTGGTGCAAC  11327490
TCCAAAGAAA AGTAACAAAC ATGTATTTTT CCACTACTAT AATACTGGTT  11327540
GGGGATTTTA ATTTAAATGA TTTGACCACA AAATGGCAGT TTTCTAGCCC  11327590
CAGCAGTTCA TTT
```

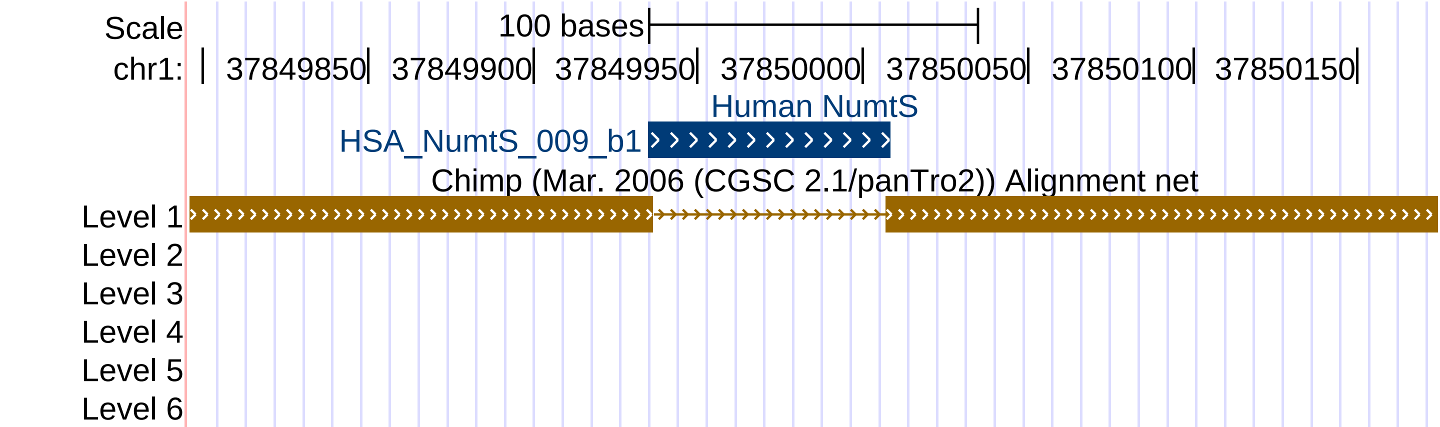

Scale
100 bases
chr1:
37849850
37849900
37849950
37850000
37850050
37850100
37850150
Human NumtS
HSA_NumtS_009_b1
Chimp (Mar. 2006 (CGSC 2.1/panTro2)) Alignment net
Level 1
Level 2
Level 3
Level 4
Level 5
Level 6

Chimp (Mar. 2006 (CGSC 2.1/panTro2)) Alignment net

View alignment details of parts of net within browser window.
Open Chimp browser at position corresponding to the part of chain that is in this window.

Type: top
Level: 1
Human position: chr1:96119-246871250
Chimp position: chr1:243305-229800167
Strand: +
Score: 17,408,357,451
Chain ID: 1
Bases aligning: 196,778,347
Chimp bases duplicated: 3,404,146
Human N's: 22,150,000 (9.0%)
Chimp N's: 12,376,710 (5.4%)
Human tandem repeat (trf) bases: 3,592,439 (1.5%)
Chimp tandem repeat (trf) bases: 3,067,464 (1.3%)
Human RepeatMasker bases: 109,429,341 (44.3%)
Chimp RepeatMasker bases: 104,856,717 (45.7%)
Human size: 246,775,132
Chimp size: 229,556,863

Fields above refer to entire chain or gap, not just the part inside the window.

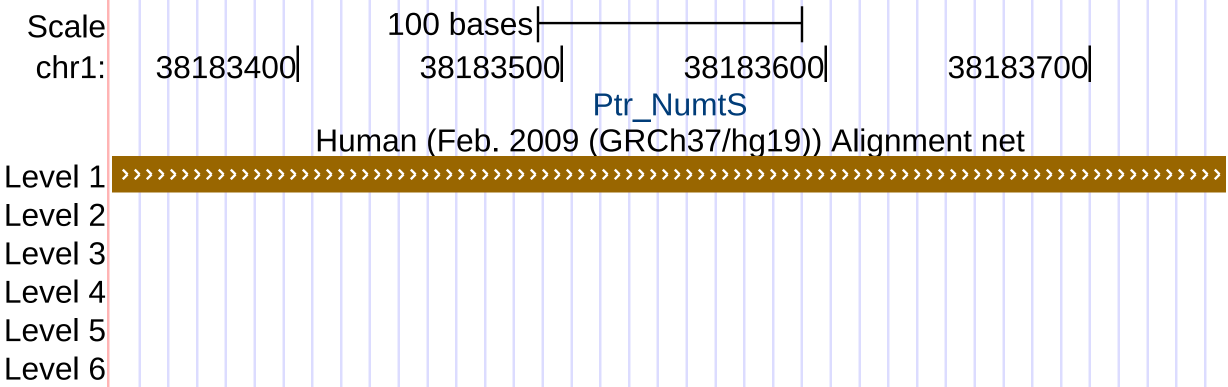
Scale
100 bases
chr1:
38183400
38183500
38183600
38183700
Ptr_NumtS
Human (Feb. 2009 (GRCh37/hg19)) Alignment net
Level 1
Level 2
Level 3
Level 4
Level 5
Level 6

Human.chr1

```
CTCCTCCCTG GAAACCTAGC ACCTAGTCCA GAGCCTGGCA CACACTTCAG  38077187
AATGCCACAA GGAGTTATTC CATCCACTGC CCTGCCTTTG GGCAGGATTG  38077237
TCCCCAACCA TCACTGGAAG CCTACGCACT CCCTTCCCCT GGCAGTGCCA  38077287
CTAGCCCTTC AGTATATAGA TTCTGCCTCT CCCAGATGGA AAGTCCTCCT  38077337
TTATAGCTGA CCttatcccc atactagtta ttatcgaaac catcagccta  38077387
ctcattcaac caatagccct ggccgtacgc ctaAGAATCC CTCCTGCTGC  38077437
AAGATGGCCG GGGCCCTTTC TCCCgTCCTT TAAGGGGAAT TACAGCAGCA  38077487
CTTCTCAaAT TTGGgTAACA TGCCAATCAT CAGGAATCTT AAAATTTAGA  38077537
CTCTGGCTca cCAGGTCTGG AATGAGACCT GAGATTCTCA TTCCTAACAA  38077587
GCTGCCCTGT GACAACACTG CTGCCGGGCC ATGCTCTGAG TAACA
```

Chimp.chr1 :

```
CTCCTCCCTG GAAACCTAGC ACCTAGTCCA GAGCCTGGCA CACACTTCAG  38183380
AATGCCACAA GGAGTTATTC CATCCACTGC CCTGCCTTTG GGCAGGATTG  38183430
TCCCCAACCA TCACTGGAAG CCTACGCACT CCCTTCCCCT GGCAGTGCCA  38183480
CTAGCCCTTC AGTATATAGA TTCTGCCTCT CCCAGATGGA AAGTCCTCCT  38183530
TTATAGCTGA CCAGAATCCC TCCTGCTGCA AGATGGCCGG GGCCCTTTCT  38183580
CCCaTCCTTT AAGGGGAATT ACAGCAGCAC TTCTCAcATT TGGaTAACAT  38183630
GCCAATCATC AGGAATCTTA AAATTTAGAC TCTGGCTCAG GTCTGGAATG  38183680
AGACCTGAGA TTCTCATTCC TAACAAGCTG CCCTGTGACA ACACTGCTGC  38183730
CGGGCCATGC TCTGAGTAAC A
```
